# Developmental Trajectories of Sleep Problems from Childhood to Adolescence Both Predict and Are Predicted by Emotional and Behavioral Problems

**DOI:** 10.3389/fpsyg.2016.01874

**Published:** 2016-12-01

**Authors:** Biyao Wang, Corinna Isensee, Andreas Becker, Janice Wong, Peter R. Eastwood, Rae-Chi Huang, Kevin C. Runions, Richard M. Stewart, Thomas Meyer, L. G. Brüni, Florian D. Zepf, Aribert Rothenberger

**Affiliations:** ^1^Department of Child and Adolescent Psychiatry and Psychotherapy, University Medical Center of GoettingenGoettingen, Germany; ^2^Centre and Discipline of Child and Adolescent Psychiatry, Psychosomatics and Psychotherapy, The University of Western Australia, PerthWA, Australia; ^3^Centre for Sleep Science, School of Anatomy, Physiology and Human Biology, The University of Western Australia, PerthWA, Australia; ^4^Telethon Kids Institute, PerthWA, Australia; ^5^Department of Psychosomatic Medicine and Psychotherapy, German Centre for Cardiovascular Research, University of GoettingenGoettingen, Germany; ^6^Child and Adolescent Psychiatry, Psychiatry Services ThurgauFrauenfeld, Switzerland; ^7^Specialised Child and Adolescent Mental Health Services, Department of Health, PerthWA, Australia

**Keywords:** sleep problems, childhood and adolescence, *latent trajectory classes*, anxiety/depression, attention problems, aggressive behavior, CBCL, Raine study

## Abstract

Although the prevalence rates of sleep disorders at different stages of childhood and adolescence have been well established, little is known about the developmental course of general sleep problems. This also holds true for the bidirectional relationship between sleep problems and emotional as well as behavioral difficulties. This longitudinal study investigated the general pattern and the latent trajectory classes of general sleep problems from a large community sample aged 5–14 years. In addition, this study examined the predictive value of emotional/behavioral difficulties (i.e., anxiety/depression, attention problems, and aggressive behavior) on sleep problems latent trajectory classes, and vice-versa. Participants (*N* = 1993) were drawn from a birth cohort of Western Australian children born between 1989 and 1991 who were followed until 14 years of age. Sleep problems were assessed at ages 5, 8, 10, and 14, respectively, whereas anxiety/depression, attention problems, and aggressive behavior were assessed at ages 5 and 17 years. Latent growth curve modeling revealed a decline in an overall pattern of sleep problems during the observed 10-year period. Anxiety/depression was the only baseline factor that predicted the longitudinal course of sleep problems from ages 5 to 14 years, with anxious and depressed participants showing faster decreasing patterns of sleep problems over time than those without anxiety or depression. Growth mixture modeling identified two classes of sleep problem trajectories: *Normal Sleepers* (89.4%) and *Troubled Sleepers* (10.6%). Gender was randomly distributed between these groups. Childhood attention problems, aggressive behavior, and the interaction between gender and anxiety/depression were significantly predictive of membership in the group of *Troubled Sleepers*. Group membership in *Troubled Sleepers* was associated with higher probability of having attention problems and aggressive behavior in mid-adolescence. Boys and girls with behavioral difficulties, and girls with emotional difficulties were at increased risk of having sleep problems during later childhood and adolescence. Developmental trajectories of sleep problems were also predictive of behavioral difficulties in later life. Findings from this study provide empirical evidence for the heterogeneity of sleep problems and their development, and emphasize the importance of understanding sleep problems and their relationship to children and adolescents’ mental health.

## Introduction

Sleep problems in children and adolescents are common (Kahn et al., 1989; Owens et al., 2000b; Goodnight et al., 2007). Estimates of the prevalence of sleep problems vary depending upon differences in definitions and methods of assessment (O’Callaghan et al., 2010). Furthermore, these disturbances can take many forms, including dyssomnias (such as insomnia) and parasomnias (such as sleep walking). Moreover, the classification of such disorders varies, depending on the system being followed – the fifth edition of the Diagnostic and Statistical Manual of Mental Disorders (DSM-5, American Psychiatric Association, 2013) or the third edition of the International Classification of Sleep Disorders (ICSD-III, American Academy of Sleep Medicine, 2014). Sleep problems often co-occur with a wide range of psychiatric and neurodevelopmental problems like autism (Allik et al., 2006; Richdale and Schreck, 2009; Cohen et al., 2014; Brand et al., 2015a), epilepsy (Pereira et al., 2012; Carotenuto et al., 2014), and tic disorders (Kirov et al., 2007; Ghosh et al., 2014).

Sleep problems not only affect children and adolescents’ psychological functioning as well as their academic, neurocognitive, and behavioral performance, but can also have a negative impact on family functioning and the well-being of family members (Curcio et al., 2006; Kheirandish and Gozal, 2006; Mitchell and Kelly, 2006; Martin et al., 2007; Meltzer and Mindell, 2007; Bell and Belsky, 2008; Kalak et al., 2014; Brand et al., 2015b). However, many parents tend to overlook or ignore sleep problems, or possibly underestimate the importance of healthy sleep habits for good daily psychological functioning of their children (Stein et al., 2001). Disturbed sleep during childhood seems to be an invisible phenomenon to many parents, and often fails to receive attention until it interferes with the child’s well-being (Smaldone et al., 2007). A greater understanding of the nature and development of sleep problems and their relationship to children’s mental health may provide critical information for researchers and clinicians in the early screening, prevention, and treatment of sleep problems in childhood and adolescence.

### Developmental Course of Sleep Problems

Although much has been learned about the prevalence rates of sleep disorders at different stages of childhood and adolescence (Ohayon et al., 2000; Ipsiroglu et al., 2002; Spruyt et al., 2005; Roberts et al., 2011; Schlarb et al., 2015), our knowledge concerning the individual development of sleep problems is still rather limited. According to a recent review of the latest science in sleep problems (Gregory and Sadeh, 2016), there is a critical need to clarify the nature and impact of such problems on child development.

To date, longitudinal studies on sleep problems during childhood and adolescence are dominated by designs with short time frames, typically using just a few years (Gregory and O’Connor, 2002). For example, Laberge et al. (2001) analyzed data on sleep problems from a randomly stratified, proportional sample of 1146 children (48.7% girls), with a particular focus on mothers’ annual report of children’s sleep patterns, sleep habits and sleep disturbances over a course of 4 years. They detected that the proportion of children with difficulties in initiating sleep declined significantly from ages 10 to 13 years. However, such studies focused on the percentage of participants with sleep problems can provide only limited information on the developmental time-course of sleep problems.

To our knowledge, very few studies have examined the development of sleep problems during childhood and adolescence in terms of a longitudinal course covering a longer time period. Gregory and O’Connor (2002) investigated the sleep problems among 490 children (46.3% girls) from ages 4 to 15 years. A decrease in the average level of sleep problems (50% decline) was found from preschool to mid-adolescence, with modest stability (*r* = 0.29). Developmental changes in sleep problems were assessed using repeated measures analyses, whilst inter-participant stability across this particular time period was assessed with bivariate correlations. Such correlational approaches tell us little about the developmental course of sleep problems; developmental trajectory analyses are required. Friedman et al. (2009) modeled the longitudinal trajectories of seven specific sleep problems frequently observed in childhood (i.e., nightmares, talks or walks, wets bed, sleeps less, sleeps more, trouble sleeping, and overtired) among 916 twins (50.8% girls) from age 4 to 16. Using latent growth curve analyses, they detected that most reported sleep problems declined over time. These studies presented the first attempt to document the average course of sleep problems (general or specific) in a community sample. However, the variable-centered approach applied in these studies precluded the possibility of examining heterogeneity in the development of sleep problems, which can provide critical information for the screening of high-risked individuals, as well as for the design of pertinent treatment approaches. Hence, person-centered trajectory analyses are required to better clarify the development of sleep problems.

Using such person-centered approaches, several studies have detected distinct subgroups of sleep problem development amongst normative child and adolescent samples. Touchette et al. (2007) investigated sleep duration among 1492 children in early childhood (2.5–6 years). Four developmental sleep duration patterns were identified: short persistent (<10h/night; 6.0%), short increasing (4.8%), 10-h persistent (50.3%), and 11-h persistent (38.9%). Similarly, Magee et al. (2014) found four distinct subgroups using growth mixture modeling (GMM): typical sleepers (40.6%), initially short sleepers (45.2%), poor sleepers (2.5%), and persistent short sleepers (11.6%) when examining the sleep duration patterns from age 0 to 1 years to age 6–7 years among 2926 children (42.7% girls). Seegers et al. (2011) examined the time spent in bed from age 10 to 13 among 1916 preadolescents (47.2%) girls and identified three distinct developmental course of time-in-bed: short sleepers (14.5%), 10.5-h sleepers (68.2%), and 11-h sleepers (17.3%). These studies identified distinct developmental courses of several normative aspects of sleep (e.g., sleep duration, time in bed) – in general about 10–15% of children were characterized as having a short sleep duration. However, to our knowledge, no studies have investigated longitudinal trajectories of general sleep problems.

Specifically, there is a need for research that examines developmental trajectories spanning childhood through adolescence. Puberty brings maturational changes to neural architecture involved in sleep as well as (in many cultures) new norms and expectations regarding sleep patterns (Dahl and Lewin, 2002; Soffer-Dudek et al., 2011). These changes (related to neural system’s reorganization) have been posited to results in increased fatigue in adolescence (Soffer-Dudek et al., 2011). Thus, sleep trajectories beginning in early childhood and spanning late adolescence are worth investigating.

### Predictors and Predictive Value of Sleep Problems

Sleep problems have been linked to both emotional (e.g., anxiety and depression) and behavioral (e.g., attention and conduct) difficulties in childhood and adolescence (Gregory and O’Connor, 2002). Improved understanding of the co-occurrence and longitudinal associations between these difficulties could potentially facilitate the development evidence-based prevention and intervention programs (Gregory and Sadeh, 2016).

The relationship between sleep and emotional problems has been examined in studies that have separated symptoms of anxiety and depression and in studies that have combined anxiety and depression into a single construct (Gregory and Sadeh, 2012). For instance, one study showed that troubled sleeping was associated with parent-reported anxiety/depression measures in children at ages 6 and 11 years (Johnson et al., 2000). Another study reported that sleep problems at age 4 were predictive of anxiety/depression in mid-adolescence (Gregory and O’Connor, 2002). Furthermore, robust associations between sleep problems and anxiety, as well as sleep problems and depression have been detected in children and adolescents (Ivanenko et al., 2005; Lovato and Gradisar, 2014; Peterman et al., 2015; see Willis and Gregory, 2015 for review). It is likely that the nature of any relationships between these psychopathologies are both *complex* and *bidirectional*. For example, it remains to be established whether sleep problems serve as a precursor to emotional difficulties (Alvaro et al., 2013; Lovato and Gradisar, 2014), or whether emotional difficulties may contribute to the development of sleep problems. Longitudinal studies have shown mixed results. Some studies have suggested that both disorders contribute similarly to the development of sleep problems (e.g., Kaneita et al., 2009; Meijer et al., 2010). However, others have suggested that the observed cause-effect associations were distinct (Ohayon and Roth, 2003; Johnson et al., 2006). Therefore, the etiological relationship between sleep and emotion remains unclear. A dearth of longitudinal, experimental, and more methodologically rigorous research limits our capacity to interpret the current literature (Peterman et al., 2015). Additional studies (in particular longitudinal ones) are required to further delineate the association between sleep and emotional problems in children and adolescents (Leahy and Gradisar, 2012).

Although most research has focused on sleep problems in association with emotional difficulties, there is emerging evidence that sleep problems may also be linked to subsequent behavioral difficulties (Gregory et al., 2008). The links between sleep and attention deficit hyperactivity disorder (ADHD) have been extensively studied (see Sadeh et al., 2006; Owens, 2008; Cortese et al., 2009; Tsai et al., 2016 for review). In this context, the topic of ADHD and related symptoms and behaviors has received considerable scientific and clinical attention (Gregory and Sadeh, 2012). In non-clinical samples, sleep disorders may affect children, and may potentially have an effect on daytime functioning of the child, including the regulation of attention (O’Callaghan et al., 2010). Longitudinal studies have suggested that sleep and attention problems are positively related. Sleep problems in early childhood are an indicator of subsequent attention problems that may persist into adolescence and adulthood (Gregory and O’Connor, 2002; Gregory et al., 2008; O’Callaghan et al., 2010; Simola et al., 2014).

Other behavioral problems such as aggression have received less attention, although there are indications that such problems may also be linked to sleep problems (Gregory and Sadeh, 2012). Several studies have suggested that poor sleep may be a causal factor in aggression and violence (see Kamphuis et al., 2012 for a review). For example, children at high-risk for sleep disorders (e.g., breathing problems, periodic leg movements during sleep) have significantly increased parent-reported aggression (Chervin et al., 2003). Children rated as having a conduct problem by a parent or teacher showed more disordered-breathing during sleep, and sleepiness predicted their behavior problems (O’Brien et al., 2011). Persistent sleep problems also appear to confer increased risk of aggressive symptoms (Pakyurek et al., 2002; Simola et al., 2014). In addition, parent-rated sleep problems in childhood are correlated with higher scores on an aggressive behavior scale later in life (Gregory et al., 2008).

Few studies have examined longitudinal relationships between sleep and behavioral problems (Gregory and Sadeh, 2012). In one such study, Pieters et al. (2015) showed that, in the short term (i.e., within a year), sleep problems appear to predict externalizing problems in early adolescence. Longer-term evidence is rarer and in the only study to date Wong et al. (2009) have suggested that early childhood sleep problems (at ages 3–8 years) predict trajectories of externalizing problems, with such problems particularly linked to sleep problems for boys.

Finally, as most research on gender differences in sleep has been conducted in adults, the literature regarding the role of gender on sleep problems in childhood and adolescence is scarce and has shown mixed results (see Krishnan and Collop, 2006; Galland et al., 2012; Mong and Cusmano, 2016 for review). Some studies have indicated that gender has no or relatively little influence on sleep (Voderholzer et al., 2003; Chaput and Tremblay, 2007; Dollman et al., 2007). In contrast, other studies have suggested the presence of gender differences with regards to sleep patterns and insomnia prevalence, the latter showing a considerable female preponderance (Hysing et al., 2013). For example, nightmares were reported more frequently by girls (Liu et al., 2000), and an increase in a variety of sleep problems (insomnia, daytime tiredness, and insufficient sleep) has been associated with the pubertal development period in girls, but not in boys (Knutson, 2005). Therefore, specific attention should be paid to the role of gender when examining sleep problems in minors.

### The Present Study

The present study aimed to investigate the overall pattern and the latent trajectory classes of general sleep problems from ages 5 to 14 years among a large community sample, using latent growth curve modeling and GMM. These analytic approaches allow examination of the overall course of sleep problems during childhood to adolescence, as well as examination of subgroups with distinct developmental patterns. The purported bidirectional nature of any relationships between sleep problems and emotional (anxiety and depression) and behavioral (attention problems and aggressive behavior) difficulties were examined by testing whether baseline emotional and behavioral problems could serve as predictors of sleep trajectory classes. Moreover, the predictive value of sleep trajectory classes on anxiety/depression, attention problems, and aggressive behavior later in life (i.e., at age 17) was analyzed. Based on extant research (Gregory and O’Connor, 2002), we expected a general decline in sleep problems over the 10-year period of the trajectories (from ages 5 to 14 years).

Further, we expected to find at least two subgroups with a distinct trajectory of sleep problems, i.e., one group including the majority of children and adolescents reporting none or few sleep problems, and another group of children and adolescents reporting persistent sleep problems during childhood and adolescence. As associations between sleep problems and emotional and behavioral difficulties have been well established (O’Callaghan et al., 2010; Kamphuis et al., 2012; Lovato and Gradisar, 2014), we expected that anxiety/depression, attention problems, and aggressive behaviors would predict the development of sleep problems, and vice-versa.

With respect to the role of gender, we did not have a specific hypothesis concerning the gender differences in sleep problems due to minimal prior research in this area (Galland et al., 2012), therefore an explorative approach was implemented for this particular research question. However, as girls tend to report more emotional difficulties (see Hyde et al., 2008; McLean and Anderson, 2009 for review), and boys tend to report more behavioral difficulties (see Archer, 2004; Rucklidge, 2010 for review), we also explored the potential moderating role of gender on the relationship between sleep problems and emotional and behavioral difficulties.

## Materials and Methods

### Participants and Procedures

Participants were from the Western Australian Pregnancy Cohort (Raine) Study. The methodology and recruitment for this study are described in detail elsewhere (Newnham et al., 1993). In brief, 2900 women between 16 and 20 weeks gestation (mean 18 weeks) were recruited from the public antenatal clinic at King Edward Memorial Hospital (KEMH) in Perth, Western Australia and surrounding private clinics between May 1989 and November 1991. Data collection occurred in accordance with Australian National Health and Medical Research Council (NHMRC) Guidelines for Ethical Conduct and was approved by the ethics committees of KEMH, Princess Margaret Hospital for Children and the University of Western Australia. Written parental consent was obtained at recruitment and at each follow-up until the age of 18. Assent was obtained from participants at age 14–17, and written consent from participants from age 18. Eligible women were required to have sufficient English-language skills to give informed consent, an expectation to deliver at KEMH, and an intention to reside in Western Australia to enable future follow-ups of their child.

Of the 2900 women enrolled, 2804 delivered live babies. There were 64 multiple births, and as such, the initial cohort consisted of 2868 children (49.3% girls). These children were assessed at birth, and were followed up at ages 1, 2, 5, 8, 10, 14, 17, 20, and 22 years of age using questionnaires and physical assessments. This study focused on the 5, 8, 10, and 14-year follow-ups, as detailed sleep problems data were collected at these assessments with adequate retention rate.

Data on sleep problems were available for 2116 participants at age 5 (73.8% retention); 2037 participants at age 8 (71.0% retention); 1994 participants at age 10 (69.5% retention); and 1774 participants at age 14 (61.9% retention). In order to better capture the developmental patterns, we focused on participants who had data on sleep problems for at least three out of the four measurement points. The effective sample size was 1993 (48.6% girls; 69.5% retention).

### Measures

#### Sleep Problems

Six items from the parent-report of Child Behavior Checklist (CBCL, Achenbach, 1991a) comprised of a ‘sleep problem scale’ and were used to measure child and adolescent sleep problems. Although not a standardized CBCL scale, the CBCL sleep composite has been shown to be strongly correlated with the validated Children’s Sleep Habits Questionnaire (CSHQ, Owens et al., 2000c) and also with clinical diagnoses of sleep disorders. The sleep composite shows similar external correlations with youths’ social problems and psychopathology symptoms as the CSHQ score (Becker et al., 2015). It has been widely used in previous research as a measure of overall sleep functioning (Stoléru et al., 1997; Gregory and O’Connor, 2002; Alfano et al., 2006; Beebe et al., 2007; Gregory et al., 2008; Storch et al., 2009; Troxel et al., 2013). The specific sleep-content items are “trouble sleeping,” “nightmares,” “overtired without good reason,” “sleeps less than most kids,” “talks or walks in sleep,” and “sleeps more than most kids during day and/or night.” Each item is rated on a 3-point scale (0 = not true, 1 = somewhat or sometimes true, 2 = very true or often true). A sum score of the 6-items scale was used to represent the level of children and adolescents’ sleep problems (range 0–12), with higher scores indicating higher levels of sleep problems. Cronbach’s alpha of the four measurement points ranged between 0.55 and 0.61

#### Emotional and Behavioral Problems at Baseline (Age 5)

The Anxious/Depressed, Attention Problems, and Aggressive Behavior scales were examined using the parent-report of CBCL (Achenbach, 1991a). None of these scales included sleep problems. The Anxious/Depressed scale consists of 14 items, sample items of the subscale included “feels or complains that no one loves him/her” and “too fearful or anxious.” The Attention Problems scale consists of 11 items, sample items of the subscale included “daydreams or gets lost in his/her thoughts” and “can’t sit still, restless, or hyperactive.” The Aggressive Behavior scale consists of 20 items, sample items of the subscale included “cruelty, bullying, or meanness to others” and “destroys things belonging to his/her family or others.” Responses were rated on a 3-point scale (0 = not true, 1 = somewhat or sometimes true, 2 = very true or often true). A sum score of all the items of the subscale was used to represent the level of children and adolescents’ emotional and behavioral problems (range 0–28, 22, 40 for the Anxious/Depressed, Attention Problems, and Aggressive Behavior scale, respectively), with higher scores indicating higher levels of emotional or behavioral problems. Cronbach’s alpha was 0.97, 0.96, and 0.98 for the Anxious/Depressed, Attention Problems, and Aggressive Behavior scale, respectively.

#### Emotional and Behavioral Problems at Age 17

The Anxious/Depressed, Attention Problems, and Aggressive Behavior scales were obtained using the Youth Self-Report (YSR, Achenbach, 1991b). Items are scored in the same way as for the parent-report CBCL. Responses were rated on a 3-point scale (0 = not true, 1 = somewhat or sometimes true, 2 = very true or often true). The sum score of all the items of the subscale was used to represent the level of children and adolescents’ emotional and behavioral problems (range 0–28, 22, 40 for the Anxious/Depressed, Attention Problems, and Aggressive Behavior scale, respectively), with higher scores indicating higher levels of emotional or behavioral problems. Cronbach’s alpha was 0.90, 0.85, and 0.92 for the Anxious/Depressed, Attention Problems, and Aggressive Behavior scale, respectively.

### Statistical Analyses

After presenting children and adolescents’ sleep problems and emotional/behavioral difficulties in a descriptive manner, the data analysis proceeded in five consecutive steps. In step 1, we applied latent growth curve modeling (LGM) to examine the overall pattern of sleep problems. Four measurements of sleep problem data assessed from childhood (age 5 years) to adolescence (age 14 years) were used as outcome variables to estimate latent growth factors that represent the average initial level (i.e., intercept) and the average growth (i.e., slopes) of sleep problems. Individual differences were modeled using random effects around these latent growth factors. A series of unconditional LGMs including different growth factors (e.g., intercept-only, linear slope, quadratic slope) were estimated to identify the model that best fit the average sleep problems longitudinal course. Because the χ^2^ is sensitive to sample size, we assessed fit primarily with the comparative fit index (CFI), root-mean-square error of approximation (RMSEA), and standardized root-mean-square residual (SRMR) using criteria of CFI ≥ 0.95, RMSEA ≤ 0.06, and SRMR ≤ 0.08 as indicators of good fit (Hu and Bentler, 1999).

In step 2, baseline emotional and behavioral predictors (i.e., anxiety/depression, attention problems, and aggressive behavior) were added to the best fitting model identified at step 1 to get a conditional LGM, which simultaneously examined the effects of baseline emotional and behavioral predictors on the course of sleep problems. The latent growth factors (e.g., intercept and slope) were regressed on the baseline predictors. The interaction effects between baseline predictors and gender on sleep problems latent growth factors were also explored.

In step 3, we used GMM to estimate latent trajectory classes in sleep problems. GMM captures the heterogeneity in unobserved subpopulations by allowing for the differences in growth parameters, and identifies a finite number of subgroups of individuals following different developmental courses (Jung and Wickrama, 2008). GMM results in separate growth model for each latent class, which differs in terms of intercept (initial level), slope (average growth), and with their unique estimates of variances and covariate influences.

A series of models were fitted, beginning with a one trajectory model and moving to a five trajectory model. Each model was fitted using at least 1000 random perturbations of starting values to ensure replication of the best likelihood and to avoid local maxima. Evaluation of the best fitting models took into account several criteria (Nylund et al., 2007). Firstly, Bayesian Information Criterion (BIC) and adjusted BIC (aBIC) were examined with lower (i.e., closer to 0) value indicating better balance between model fit and parsimony. Secondly, Lo–Mendell–Rubin likelihood ratio test (LMR-LRT) and bootstrap likelihood ratio test (BLRT) were used to compare the k and the k-1 trajectory models. A significant *p*-value (<0.05) in LMR-LRT and BLRT indicated a statistically significant improvement in model fit with the inclusion of one more trajectory. Thirdly, we examined the entropy index that ranges from 0 to 1 while entropy closer to one indicates better classification.

In step 4, participants were assigned to their most likely trajectory latent class based on their highest posterior probability. Gender-specific group membership was compared. We conducted multinomial logistic regression with baseline emotional and behavioral predictors (i.e., anxiety/depression, attention problems, and aggressive behavior) as independent variables to examine their usefulness in predicting sleep problems trajectory group membership. The interaction between gender and each predictor variable was also examined. Odds ratios with 95% confidence intervals were estimated and reported.

In step 5, to test the predictive value of sleep problems trajectory classes on emotional and behavioral problems later in adolescence, we examined level of later anxiety/depression, attention problems, and aggressive behavior conditional on the latent trajectory class membership. Mean scores of anxiety/depression, attention problems, and aggressive behavior at age 17 years were compared between sleep problem trajectory classes using independent sample *t*-test.

Latent growth curve modelings and growth mixture modelings were performed using Mplus version 7.3 (Muthén and Muthén, 1998-2015). Other analyses were carried out using SPSS version 23. Missing data on the outcome variables were handled through full information maximum likelihood (FIML) estimation in Mplus as a standard procedure under the assumption of missing at random (Muthén and Muthén, 1998-2015). Individuals with missing values on predictors were excluded from multinomial logistic regression and comparison of means.

## Results

### Descriptive Statistics

**Table 1** presents descriptive statistics and bivariate correlations for all study variables. Results from Little’s MCAR test showed that missing data on all variables were missing completely at random (*p* = 0.317). Means of children and adolescents’ sleep problems experienced a significant decrease over time (*t* = 11.855, *df* = 1576, *p* < 0.001). Almost all study variables were significantly inter-correlated (except for anxiety/depression at age 5 and attention problems at age 17). Correlations between sleep problems measured in the four waves showed that the magnitude of associations between adjacent assessments (*r*s = 0.50–0.53, *p* < 0.01) was greater than that between non-adjacent ones (*r*s = 0.38–0.43, *p* < 0.01). Cross-sectional relationships between sleep problems and baseline emotional/behavioral predictors (*r*s = 0.41–0.46, *p* < 0.01) were also higher than their relationships over time (*r*s = 0.23–0.34, *p* < 0.01). The longitudinal relationships between sleep problems (at age 5, 8.10, and 14) and later emotional/behavioral (at age 17) was trivial (*r*s = 0.09–0.16, *p* < 0.01).

**Table 1:** Descriptive statistics and bivariate correlations between all study variables.

	*N*	*M*	*SD*	1	2	3	4	5	6	7	8	9	10
(1) Sleep problems at 5	1857	1.30	1.49	1									
(2) Sleep problems at 8	1861	1.21	1.55	0.52^∗∗^	1								
(3) Sleep problems at 10	1902	1.01	1.40	0.42^∗∗^	0.53^∗∗^	1							
(4) Sleep problems at 14	1705	0.85	1.34	0.38^∗∗^	0.43^∗∗^	0.50^∗∗^	1						
(5) Anxiety/depression at 5	1873	2.78	2.93	0.46^∗∗^	0.30^∗∗^	0.25^∗∗^	0.24^∗∗^	1					
(6) Attention problems at 5	1860	2.88	2.79	0.41^∗∗^	0.30^∗∗^	0.27^∗∗^	0.23^∗∗^	0.49^∗∗^	1				
(7) Aggressive behavior at 5	1789	8.08	6.21	0.43^∗∗^	0.34^∗∗^	0.30^∗∗^	0.29^∗∗^	0.53^∗∗^	0.58^∗∗^	1			
(8) Anxiety/depression at 17	1182	5.04	5.20	0.11^∗∗^	0.10^∗∗^	0.09^∗∗^	0.14^∗∗^	0.11^∗∗^	0.06^∗^	0.10	1		
(9) Attention problems at 17	1182	5.00	3.13	0.11^∗∗^	0.10^∗∗^	0.11^∗∗^	0.16^∗∗^	0.05	0.13^∗∗^	0.13^∗∗^	0.60^∗∗^	1	
(10) Aggressive behavior at 17	1182	7.91	5.11	0.13^∗∗^	0.15^∗∗^	0.12^∗∗^	0.12^∗∗^	0.10^∗∗^	0.11^∗∗^	0.23^∗∗^	0.46^∗∗^	0.60^∗∗^	1


*The total sample size was 1993. ^∗^*p* < 0.05, ^∗∗^*p* < 0.01.*

### Longitudinal Course of Sleep Problems from Age 5 to 14

#### Unconditional Latent Growth Curve Models

**FIGURE 1:**
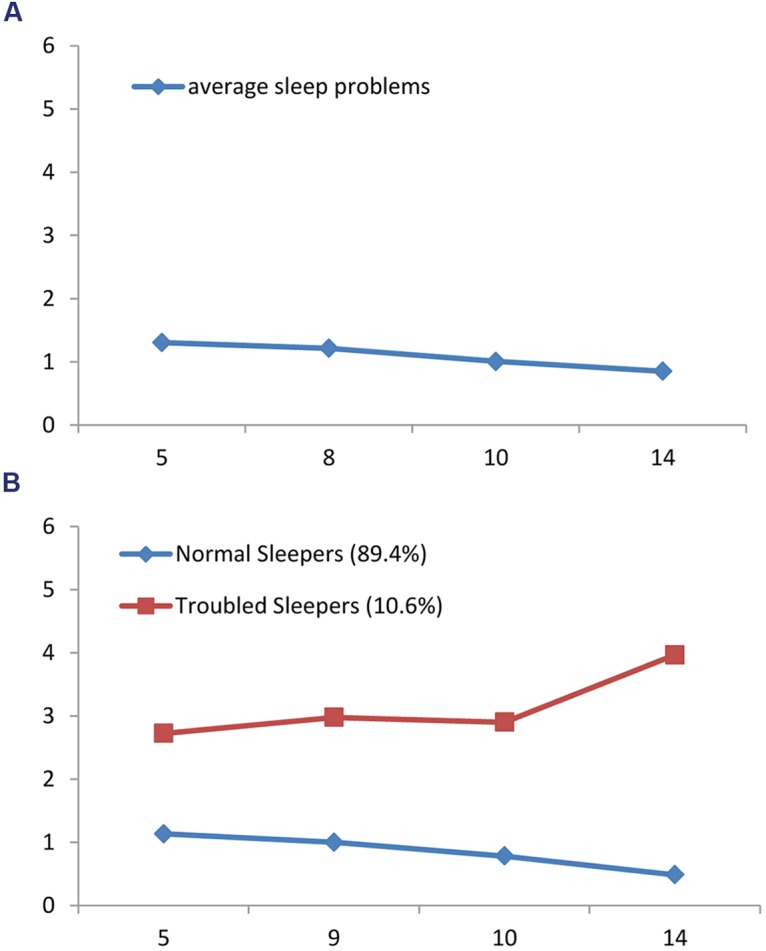
**(A)** General course of sleep problems from age 5 to 14. **(B)** Latent trajectory classes of sleep problems from age 5 to 14.

A model including a linear slope (CFI = 0.85, RMSEA = 0.13, and SRMR = 0.08) fit the data better than an intercept-only model (CFI = 0.98, RMSEA = 0.07, and SRMR = 0.03). The addition of a quadratic slope also improved the model fit (CFI = 0.99, RMSEA = 0.06, and SRMR = 0.01). Thus, a model including both linear and quadratic slopes was selected for the subsequent analyses. A significant negative linear slope (*s* = -0.56, *p* < 0.01) and a non-significant quadratic slope (*q* = 0.04, *p* = 0.76) indicated a stable decreasing trend over time (**Figure 1A**). Significant variances were observed around the intercept and both slope factors, suggesting inter-individual differences in sleep problems at initial level as well as in the development of sleep problems. This significant variance suggested that GMM might provide insight into the heterogeneity in the development of sleep problems.

#### Baseline Predictors of Longitudinal Course of Sleep Problems

Results from the conditional LGM are presented in **Table 2**. Significant effects were observed on the intercept factor for all baseline emotional and behavioral predictors. Children and adolescents with higher levels of anxiety/depression, attention problems, and aggressive behavior at baseline were concurrently reported with higher levels of sleep problems. Gender was not significantly related to the initial level of sleep problems.

**Table 2:** Unstandardized parameter estimates for predictors of sleep problems latent growth factors.

Predictors	Intercept	Linear slope	Quadratic slope
			
	Estimate (*SE*)	Estimate (*SE*)	Estimate (*SE*)
Gender	0.10 (010)	0.12 (0.45)	-0.16 (0.46)
Anxiety/depression at 5	0.15 (0.02)^∗∗^	-0.35 (0.10)^∗∗^	0.23 (0.09)^∗∗^
Attention problems at 5	0.10 (0.02)^∗∗^	-0.14 (0.10)	0.10 (0.10)
Aggressive behavior at 5	0.04 (0.01)^∗∗^	0.04 (0.05)	-0.04 (0.05)


*Gender was dummy-coded as boys = 0 and girls = 1.*

*^∗∗^*p* < 0.01.*

Only emotional difficulties at age 5 were predictive of the linear and quadratic slope factors. Anxious and depressed children showed a faster decreasing pattern of sleep problems over time, characterized by a steeper and a subsequent more marked decline. Conversely, gender and baseline differences in behavioral vulnerabilities (i.e., attention problems and aggressive behavior) did not predict the longitudinal course of sleep problems from age 5 to 14.

With respect to the moderating role of gender, no significant interaction effect between gender and anxiety/depression, attention problems, or aggressive behavior was found (effect not presented). Associations between emotional and behavioral predictors and the development of sleep problems were similarly affecting boys and girls.

### Latent Trajectory Classes of Sleep Problems from Age 5 to 14

#### Unconditional Growth Mixture Models

Unconditional GMMs with 1–5 trajectory classes were estimated including both linear and quadratic slopes. **Table 3** shows the model selection criteria used to decide on the best class solution for sleep problems trajectories. The BIC and aBIC both consistently declined for 1- through 5-class solution, although with a decelerated decreasing rate with the addition of classes. However, the model complexity also increased with the number of latent classes. Both the LMR-LRT and BLRT suggested that the 2-class solution significantly improved the model fit as compared to the 1-class solution, whereas the 3-class solution did not fit the data better than the 2-class solution. Moreover, the 3-class solution yielded two classes showing a highly similar trend. In addition, the 2-class solution achieved a slightly higher entropy (0.92) than the 3-class solution (0.91), although they both showed adequate classification accuracy. Therefore, the 2-class solution was chosen.

**Table 3:** Model selection criteria to determine trajectory classes of sleep problems.

Model	Log likelihood	BIC	aBIC	LMR-LRT	BLRT	Entropy
1-class	-12188.88	24476.32	24435.02	–	–	
2-class	-11808.13	23745.41	23691.40	<0.001	<0.001	0.92
3-class	-11589.41	23338.36	23271.64	0.06	1.00	0.91
4-class	-11466.47	23122.68	23043.25	0.07	0.08	0.94
5-class	-11348.76	22917.65	22825.52	0.24	1.00	0.93


*BIC, Bayesian Information Criterion; aBIC, Sample size adjusted BIC; LMR, Lo–Mendell–Rubin test; BLRT, Bootstrap likelihood ratio test.*

The quadratic slope emerged to be significant only for one trajectory class. After fixing the non-significant quadratic slope, the new 2-class solution achieved a better model fit (BIC = 23740.00; aBIC = 23689.16), therefore this more parsimonious model is presented. Latent trajectory classes of sleep problems are shown in **Figure 1B**.

The trajectory with the majority of adolescents (89.4%) was labeled *Normal Sleepers* (*n* = 1782). This class included adolescents with a lower initial level of sleep problems (*i* = 1.16, *p* < 0.01), which tended to decrease over time, as indicated by the significant negative linear slope (*s* = -0.75, *p* < 0.01). The second class was the *Troubled Sleepers* (*n* = 211), which consisted of adolescents who followed a persistent higher sleep problems trajectory (10.6 %). This class was described by a higher initial level of sleep problems (*i* = 2.73, *p* < 0.01), and a curvilinear trend as indicated by a non-significant negative linear slope (*s* = -0.48, *p* = 0.59) and a significant positive quadratic slope (*q* = 2.08, *p* = 0.02).

A contingency analysis was performed to determine whether boys and girls were similarly distributed in the sleep problems latent trajectory classes. Results of chi-square test showed a random gender distribution between *Normal Sleepers* and *Troubled Sleepers* (χ^2^ = 0.92, *p* = 0.34).

#### Baseline Predictors of Sleep Problems Latent Trajectory Classes

Results from multivariate binomial logistic regression showing the effects of baseline emotional and behavioral predictors as well as their interaction with gender on sleep problems trajectory classes are presented in **Table 4**. Only baseline behavioral predictors were able to differentiate the sleep problems between the two trajectory classes. Specifically, children and adolescents with attention problems and aggressive behavior at age 5 years were more likely to follow the *Troubled Sleepers* trajectory class as compared to those without behavioral difficulties. Moreover, the interaction between gender and anxiety/depression was significantly predictive of sleep problems class membership, suggesting girls with emotional problems were at elevated risk of being *Troubled Sleepers.*

**Table 4:** Multivariate binomial logistic regression of baseline predictors on sleep problems trajectory classes.

Predictors	*Normal Sleepers* vs. *Troubled Sleepers*			
	
	OR	Lower 95% CI	Upper 95% CI	Sig.
Gender	2.76	1.72	6.62	0.97
Anxiety/depression at 5	2.72	2.53	2.95	0.96
Attention problems at 5	3.00	2.75	3.30	0.03
Aggressive behavior at 5	2.94	2.82	3.07	0.00
Gender^∗^Anxiety/depression at 5	3.24	2.85	3.74	0.01
Gender^∗^Attention problems at 5	2.50	2.23	2.84	0.19
Gender^∗^Aggressive behavior at 5	2.65	2.50	2.82	0.43


*Gender was dummy-coded as boys = 0 and girls = 1.*

#### Sleep Problems Latent Trajectory Classes and Later Emotional/Behavioral Problems

We examined the level of emotional and behavioral difficulties later in life among sleep problem trajectory classes. A reduced sample (*n* = 1182) was used in this step due to missing data on emotional and behavioral problems at age 17. Means of anxiety/depression, attention problems, and aggressive behavior at age 17 in the reduced sample were compared. Results from independent sample *t*-test showed that there were significant differences at the level of attention problems (*M*_Normal_ = 4.93, *M*_Troubled_ = 5.73, *p* < 0.05) and aggressive behavior (*M*_Normal_ = 7.79, *M*_Troubled_ = 9.04, *p* < 0.05) between *Normal Sleepers* and *Troubled Sleepers* at age 17. No significant differences were observed for later anxiety and depression (*M*_Normal_ = 4.96, *M*_Troubled_ = 5.81, *p* = 0.14) between the trajectory groups. Missingness analyses indicated that the participants (*n* = 811) who dropped out reported significantly higher sleep problems at age 13 (*p* < 0.05) and attention problems at age 5 (*p* < 0.05) compared with the reduced sample.

## Discussion

Viewed as a whole, sleep problems decreased stably across the childhood and adolescence period. This finding is consistent with the work of Gregory and O’Connor (2002) in a similar aged community sample (5–14 years vs. 4–15 years) of 400 Americans, despite the current study being developed and designed in a different cultural background (Australian) and using a different statistical method (LGM vs. repeated measures analyses). Both of the studies suggest that sleep problems show a gradual declining trend during childhood and adolescence in the general population. However, this group trend does not elucidate the implicit heterogeneity of these data. Indeed, this normative picture is complicated by our finding of heterogeneity in the development of sleep problems, which showed that one in 10 young people experience chronic sleep problems into adolescence.

Hence, our second major finding from GMM identified two distinct trajectory classes of sleep problems from childhood to adolescence. The majority of children and adolescents (89.4%) reported few sleep problems, which is modeled by the latent growth curve analyses and also is reflected by the overall trajectory. However, this general course obscured the small group of children and adolescents (10.6%) who were troubled with higher levels of sleep problems over the 10-year period. Notably, this group of troubled sleepers was characterized with high, but stable sleep problems from age 5 to 10 and a sudden statistically significant rise in sleep problems from age 10 to 14 years probably due to the pubertal developmental phase. In consideration of the high prevalence of sleep disturbance in the adult population (10–40% of insomnia, e.g., Ohayon, 2002; Morphy et al., 2007) and corresponding predictive value of adolescence sleep (e.g., Dregan and Armstrong, 2010), such reported sleep problems might continue into adulthood and thus reflect a persistent disturbance. However, given the lack of later-time-point data, such an assumption should remain speculative, but should be a focus of examination in future studies.

In summary, these findings provide empirical support for the existence of two distinct subgroups of children and adolescents with different levels of sleep problems over time. The presence of *Troubled Sleepers* echoes previous literature on the continuation/persistence of sleep problems (e.g., Fricke-Oerkermann et al., 2007; Luo et al., 2013).

These results may help to reconcile a paradox within the sleep literature. On the one hand, studies on the longitudinal course of general sleep problems (Gregory and O’Connor, 2002; Umlauf et al., 2011) suggest that sleep problems decrease from early childhood through adolescence. On the other hand, sleep problems amongst adolescents are considered to be especially prevalent (e.g., Pieters et al., 2015) and receive special attention (e.g., Gradisar et al., 2011). Although our mixture modeling could visualize a subgroup with declining trajectory to reflect the cessation/remission of sleep problems (the 5-class solution), we did not report it due to insufficient statistical support. As was already proved by previous studies using sleep electroencephalography and actigraphy (Hatzinger et al., 2013, 2014; Perkinson-Gloor et al., 2015), the present findings reveal that during childhood and adolescence, the long term development of sleep problems is dominated by its stability. Heterogeneous trajectory classes of sleep problems mainly differed in the level (quantity-wise), rather than the shape of developmental course (quality-wise). If the stable nature of the trajectory class of the persistent troubled sleepers can be replicated and predictors are found, we might be able to identify this group of children and adolescents from early assessments on and find ways of prevention or early intervention of sleep problems.

In this study, early *emotional and behavioral problems* were used as predictors of the general course and distinct trajectory subgroups of sleep problems. When the average course of sleep problems in the general population was considered, anxiety/depression, attention problems, and aggressive behavior (together also known as the Dysregulation Profile, see Deutz et al., 2016) at baseline all predicted higher initial levels of sleep. Furthermore, children with early attention problems or aggressive behavior, and girls with early anxiety and depression were more likely to be *Troubled Sleepers* compared to their counterparts.

These results suggest that both emotional and behavioral problems should be considered as potent risk factors of sleep problems. Also, these findings suggest that behavioral problems share a close link with the initial level, rather than the change of sleep problems over time. This suggests that accounts of the relationship of these emotional and behavioral problems and sleep should focus on the earlier years of life, prior to the fifth year. The interaction between emotional problems and gender, being predictive of group membership in *Troubled Sleepers*, may stem from girls’ greater vulnerabilities for anxiety and depression (see Hyde et al., 2008; McLean and Anderson, 2009 for review) and calls for further investigation into this complex relationship.

Trajectory group membership of *sleep problems* were used as predictors of later emotional and behavioral problems. Results showed that *Troubled Sleepers* reported significantly higher levels of attention problems and aggressive behavior at age 17 years when compared to *Normal Sleepers*, while they had similar levels of anxiety and depression. These results are partly consistent with findings from previous research (e.g., Gregory and O’Connor, 2002; Umlauf et al., 2011; Pieters et al., 2015) that sleep problems are predictive of later behavioral/emotional problems. Our findings extend these findings in important ways. For example, Gregory and O’Connor (2002) used sleep problems as single measurement at age 4 years while we used sleep problems as group membership of trajectory classes from ages 5 to 14 years. Pieters et al. (2015) examined change in relative levels via panel analyses over a single year, whereas we were able to examine over a decade of data. However, we still know little about how sleep problems are implicated in different modes of aggression. Our data, in conjunction with research indicating the impact of fatigue on impulsivity amongst adolescents (e.g., Abe et al., 2010), would suggest that future research focuses on the role of sleep disturbance in impulsively enacted aggression and violence, including cyber-aggression (Runions, 2013).

Our findings are indicative of the *bidirectional relationship between sleep problems and behavioral problems*. In contrast, only a one-way relationship was found between sleep and emotional problems, that early anxiety and depression were predictive of higher initial level of general course of sleep problems. According to the recent findings from Mulraney et al. (2016), sleep problems and emotional problems were predictors of one another in a 6-month interval but not in a 12-month interval, such a relationship might weaken or vanish in long term development.

With respect to the role of gender in sleep problems, our findings suggest that there is no or little gender difference, either in the prevalence, initial level, general course or distinct trajectory classes of sleep problems. These findings support previous studies (Voderholzer et al., 2003; Chaput and Tremblay, 2007; Dollman et al., 2007) and extend our knowledge to individual-level. The only exception was the finding that girls with emotional problems were more likely to be *Troubled Sleepers*. The underlying mechanism of such an interaction effect is unclear, however, may be an important direction for future research.

Findings from this study have *implications for the screening and treatment of sleep problems*. The stable nature of sleep problems suggests that children with sleep problems at early time point have a great chance/risk to maintain or aggravate the symptoms throughout childhood and adolescence. Thus, early screening (i.e., in kindergarten) could present a particularly important time for early intervention to improve future sleep behaviors. Such early intervention might be particularly important given the bidirectional relationship between sleep and behavioral problems. Indeed, the presence of attention problems and aggressive behavior may be suggestive of increased later sleep problems and vice-versa. It would be useful for clinicians therefore to not only assess sleep problems but also behavioral problems and vice-versa to evaluate the integrated risk. In addition, the data from our study suggests that special attention should to be paid to girls with emotional problems since they tend to show more sleep problems in childhood and adolescence.

It is important to consider *strengths and limitations of this study*. The adoption of a large sample size and longitudinal design enabled us to model the general pattern of sleep problems during childhood and adolescence and replicate the work of Gregory and O’Connor (2002). Furthermore, the utilization of GMM, a person-centered approach, allowed us to identify distinct trajectory classes in sleep problems, which is hidden in general population analyses. To our knowledge, this is the first study to empirically investigate subgroups with a different course of general sleep problems and thus find subgroup-specific predictors for better assessment and treatment.

Despite these strengths, some limitations must be considered. First, sleep problems were measured using six items derived from CBCL to assess the general sleep problems (composed of different kinds of sleep problems) and thus might not generalize to specific categorical and isolated disorders of sleep, like insomnia, hypersomnia, and parasomnia. These disorders have certainly different pathological backgrounds, implications, and trajectories. However, even when adding an exploratory factor analysis, we could not derive valid factors related to these three categories. Therefore, we suggest future study to apply more specific (or even objective) sleep assessments in longitudinal designs, to investigate the unique feature of these disorders. Second, data regarding sleep problems and early emotional/behavioral problems were based exclusively on parent-reports and there is potential for rater bias (Owens et al., 2000a; Gregory et al., 2006), although the consistency of prediction to self-reported emotional and behavioral problems at age 17 may mitigate this concern. Furthermore, since parents typically have less knowledge of internalizing problems in their adolescents, self-reports may be more appropriate than parent-reports in assessing older children. Third, although the CBCL sleep composite is a valid and reliable parameter, formal diagnoses of sleep disorder or mental health status (e.g., diagnoses of anxiety/depression disorder or other psychiatric abnormalities) would have strengthened our evaluation. Fourth, given the focus on emotional and behavioral problems, we did not examine other predictors that might influence the development of sleep problems, such as family/parental factors (Adam et al., 2007; Cousins et al., 2007; Brand et al., 2009; Kalak et al., 2012; Bajoghli et al., 2013). It would be of particular value if future research could additionally examine the predictive value of other risk factors. Finally, despite the longitudinal methodology, this is an observational study, which at best can only show associations, not causation, between trajectories and risk factors and outcomes.

## Conclusion

In summary, this study revealed a lessening overall pattern of sleep problems from childhood to adolescence. Within this general decline, two distinct trajectory classes of sleep problems were identified: *Normal Sleepers* with the great majority of children and adolescents reporting lower level sleep problems over time, and *Troubled Sleepers*, a small group of children and adolescents reporting persistently higher level of sleep problems throughout the period investigated. Children and adolescents with attention problems, aggressive behavior, and girls with anxiety/depression at age 5 years were more likely to be *Troubled Sleepers* compared with *Normal Sleepers*. Those subjects in the *Troubled Sleepers* trajectory group had higher levels of attention problems and aggressive behavior at age 17 years. This study provided evidence for the stable nature of sleep problems during childhood and adolescence and partly supported the bidirectional model between sleep and emotional/behavioral problems.

## Author Contributions

BW and AR conceived and designed the study; FZ was responsible for data acquisition; BW performed the statistical analysis, interpreted the data, drafted and revised the manuscript; CI, AB, and AR contributed to interpretation of the data, drafting and revising the paper; JW, PE, R-CH, KR, RS, TM, LB, and FZ helped to drafted and revised the manuscript. All authors read and approved the final manuscript and agree to be accountable for all aspects of the work specifically to responding to questions related to the accuracy or integrity of any part of the work. FZ and AR are joint senior author. FZ is responsible for the group’s correspondence with Raine Study.

## Conflict of Interest Statement

The authors declare that the research was conducted in the absence of any commercial or financial relationships that could be construed as a potential conflict of interest.
